# Demographics and lifestyle factors of patients with hidradenitis suppurativa: An All of Us database analysis

**DOI:** 10.1016/j.jdin.2024.08.002

**Published:** 2024-09-03

**Authors:** Gaurav N. Pathak, Timothy Makkar, Suraj S. Pathak, Shazli Razi, Cindy Wassef, Babar Rao

**Affiliations:** a Department of Dermatology, Rutgers Robert Wood Johnson Medical School, Piscataway, New Jersey; b Department of Computer Science, Manning College of Information and Computer Sciences, University of Massachusetts, Amherst, Massachusetts; c Department of Internal Medicine, Jersey Shore University Medical Center, Neptune, New Jersey; d Department of Dermatology, Rao Dermatology, Atlantic Highlands, New Jersey

**Keywords:** All of Us, comorbidities, epidemiology, hidradenitis suppurativa, HS, underrepresented groups

*To the Editor:*

Hidradenitis suppurativa (HS) is a chronic immune-mediated inflammatory skin disease characterized by hair follicle occlusion with dysregulated inflammation, healing, and scarring.^1^ The clinical presentation of HS is heterogeneous and atypical clinical presentations in skin of color (SOC) may cause diagnostic delay and lead to misdiagnosis. We characterize patterns of HS using the “All of Us” National Institutes of Health database, a large database that promotes research in health disparities and underrepresented communities.

A cross-sectional analysis of the All of Us database using the controlled tier version 7 data set was conducted. Participants were eligible for the analysis if they had demographic information linked with their electronic health record data. Patients with HS were stratified based on HS diagnosis code (SNOMED 4241223) and the characteristics/lifestyle factors of the patients populations were compared with non-HS using χ^2^ tests. Age-adjusted univariate and multivariate logistic regression were used to estimate odds ratios (ORs) of HS diagnosis for each demographic and lifestyle factor assessed. Multivariable regression controlled for all socioeconomic and lifestyle factors listed in Tables I and II.

**Table I:** Characteristics and adjusted odds ratios of demographic data for HS versus non-HS cohorts in the All of Us electronic health record

Parameter	HS *n* = 1653 (%)	Non-HS *n* = *285,359* (%)	*P* value∗	Age-adjusted OR (95% CI)	Multivariable adjusted OR (95% CI)†
Race			<.001		
White	662 (40.04)	154,016 (53.97)		Reference	Reference
Black/African American	625 (37.81)	57,639 (20.19)		2.10 (1.88-2.35)	1.87 (1.64-2.14)
Hispanic only‡	226 (13.67)	47,474 (16.63)		0.89 (0.77-1.04)	0.85 (0.62-1.17)
Asian	≤20§	8277 (2.9)		0.39 (0.24-0.63)	0.74 (0.45-1.21)
Otherװ	76 (4.59)	9995 (3.5)		1.47 (1.16-1.87)	1.36 (1.02-1.81)
Unknown¶	47 (2.84)	7958 (2.78)		—	—
Ethnicity			.171		
Not Hispanic	1307 (79.06)	220,628 (77.31)		Reference	Reference
Hispanic/Latino	278 (16.81)	53,776 (18.84)		1.11 (0.72-1.71)	1.14 (0.85-1.52)
None of these	21 (1.27)	2996 (1.04)		0.73 (0.64-0.84)	0.89 (0.53-1.49)
Unknown¶	47 (2.84)	7959 (2.78)		—	—
Age, y			<.001		
≤65	1467 (88.74)	195,865 (68.63)		Reference	Reference
>65	186 (11.25)	89,494 (31.36)		0.27 (0.23-0.32)	0.36 (0.30-0.43)
Gender identification			<.001		
Male	297 (17.96)	107,912 (37.81)		Reference	Reference
Female	1301 (78.7)	170,130 (59.61)		2.56 (2.25-2.90)	1.41 (0.82-2.43)
Nonbinary#	25 (1.51)	1962 (0.68)		3.74 (2.48-5.64)	2.03 (1.13-3.65)
Unknown¶	30 (1.81)	5355 (1.87)		2.47 (1.47-4.16)	1.44 (0.76-2.74)
Sexual orientation			<.001		
Straight	1353 (81.85)	251,033 (87.97)		Reference	Reference
Not straight	231 (13.97)	24,475 (8.57)		1.53 (1.33-1.76)	1.27 (1.09-1.48)
Unknown¶	69 (4.17)	9851 (3.45)		1.38 (1.04-1.84)	1.20 (0.89-1.62)
Sex assigned at birth			<.001		
Male	302 (18.26)	108,437 (38.0)		Reference	Reference
Female	1319 (79.79)	171,082 (59.95)		2.55 (2.24-2.89)	1.32 (0.77-2.27)
Other/unknown∗∗	32 (1.93)	5840 (2.04)		2.21 (1.36-3.61)	1.20 (0.65-2.24)
Education			<.001		
College graduate††	456 (27.58)	119,790 (41.97)		Reference	Reference
Grade 12/GED + college 1-3 y	1000 (60.49)	128,716 (45.1)		1.82 (1.62-2.03)	1.18 (1.04-1.34)
Less than HS	150 (9.07)	27,717 (9.71)		1.23 (1.02-1.48)	0.93 (0.75-1.15)
Other	47 (2.84)	9136 (3.2)		1.29 (0.90-1.85)	0.96 (0.66-1.41)
Income			<.001		
>25k	674 (40.77)	150,595 (52.77)		Reference	Reference
<25k	658 (39.8)	74,163 (25.98)		1.73 (1.55-1.93)	1.20 (1.05-1.38)
Unknown¶	321 (19.41)	60,601 (21.23)		1.13 (0.99-1.30)	1.07 (0.92-1.25)
Disability status			<.001		
No disability‡‡	336 (20.32)	75,147 (26.33)		Reference	Reference
Disability§§	205 (12.4)	27,153 (9.51)		1.78 (1.49-2.12)	0.99 (0.82-1.19)
Unknownװװ	1112 (67.27)	183,059 (64.15)		1.36 (1.20-1.54)	0.99 (0.87-1.12)
Health insurance			.004		
Yes	1534 (92.8)	258,825 (90.7)		Reference	Reference
No	65 (3.93)	16,668 (5.84)		0.52 (0.40-0.67)	0.74 (0.57-0.95)
Other	54 (3.26)	9866 (3.45)		0.85 (0.62-1.17)	0.91 (0.65-1.27)
Occupation			.035		
Employed	651 (39.38)	116,452 (40.8)		Reference	Reference
Not employed	780 (47.18)	136,297 (47.76)		1.43 (1.28-1.59)	0.92 (0.81-1.05)
Other	222 (13.43)	32,610 (11.42)		1.41 (1.20-1.65)	1.06 (0.90-1.25)

The symbol “—” represents dropped variable in age and multivariate regression calculations due to high multicollinearity.

*GED*, General educational development; *HS*, hidradenitis suppurativa; *OR*, odds ratios.

∗ *P* values correspond to the χ^2^ analysis of the respective demographic factor for HS compared with non-HS cohorts.

† Multivariable regression controlled for all socioeconomic and lifestyle factors listed in Tables I and II.

‡ Participants who selected Hispanic only for race question.

§ As per All of Us database reporting guidelines, all values under 20 must be represented as ≤20.

װ Includes more than one population, another single population, none of these, Middle Eastern/North African, and Native Hawaiian/Pacific Islander.

¶ Skip and prefer not to answer.

# Nonbinary, additional, transgender, multiselect.

∗∗ Intersex, none, no matching concept, skip, prefer not to answer.

†† College graduate and advanced degree.

‡‡ Answer to “no” to each and every disability question including deaf, blind, difficulty concentrating, difficulty walking/climbing, difficulty dressing/bathing, difficulty doing errands alone.

§§ Answered “yes” to at least one of the disability questions.

װװ Did not answer at least one of the disabilities questions.

There were 1653 patients with HS out of a total of 287,012 electronic health records (prevalence 0.58%; 95% CI, 0.55-0.60). Patients with a higher multivariable adjusted odds of HS include Blacks (OR, 1.87), women (OR, 2.03), nonbinary (OR, 2.03), patients with college education (OR, 1.18), and patients earning <25k (OR, 1.20). Patients at lower odds include uninsured (OR, 0.74), and >65 patients (OR, 0.36) (Table I). Patients with HS were significantly more likely to use tobacco, marijuana, e-cigarettes, and hookah products (*P* < .001).

**Table II:** Characteristics of lifestyle factors of HS versus non-HS cohorts in the All of Us electronic health record

Parameter	HS *n* = 1653 (%)	Non-HS *n* = 285,359 (%)	*P* value∗	Age-adjusted OR (95% CI)	Multivariable adjusted OR (95% CI)†
Smoker‡			<.001		
No	771 (46.64)	163,250 (57.2)		Reference	Reference
Yes	833 (50.39)	113,762 (39.86)		1.65 (1.50-1.83)	1.46 (1.30-1.63)
Unknown	49 (2.96)	8347 (2.92)		1.29 (0.97-1.72)	0.99 (0.71-1.38)
Alcohol drinker			.436		
No	190 (11.49)	30,069 (10.53)		Reference	Reference
Yes	1426 (86.26)	249,099 (87.29)		0.97 (0.83-1.13)	0.94 (0.79-1.11)
Unknown	37 (2.23)	6191 (2.16)		1.03 (0.72-1.48)	0.87 (0.57-1.33)
E-cigarette user			<.001		
No	1160 (70.17)	234,740 (82.26)		Reference	Reference
Yes	431 (26.07)	43,137 (15.11)		1.61 (1.44-1.81)	1.34 (1.17-1.53)
Unknown	62 (3.75)	7482 (2.62)		1.64 (1.27-2.13)	1.58 (1.13-2.21)
Marijuana user			<.001		
No	244 (14.76)	44,636 (15.64)		Reference	Reference
Yes	245 (14.82)	22,125 (7.75)		1.71 (1.43-2.05)	1.51 (1.25-1.82)
Unknown	1164 (70.41)	218,598 (76.6)		0.95 (0.83-1.10)	0.96 (0.83-1.11)
Hookah smoker			<.001		
No	1227 (74.22)	230,296 (80.7)		Reference	Reference
Yes	374 (22.62)	46,666 (16.35)		1.20 (1.06-1.35)	1.25 (1.09-1.44)
Unknown	52 (3.14)	8397 (2.94)		1.12 (0.84-1.48)	0.83 (0.57-1.22)
Cigar smoker			.016		
No	996 (60.25)	175,688 (61.56)		Reference	Reference
Yes	588 (35.57)	101,159 (35.44)		1.00 (0.90-1.11)	1.06 (0.93-1.19)
Unknown	69 (4.17)	8512 (2.98)		1.49 (1.16-1.90)	1.56 (1.14-2.15)
Smokeless tobacco user			.146		
No	1490 (90.13)	253,743 (88.92)		Reference	Reference
Yes	120 (7.25)	24,555 (8.6)		0.74 (0.62-0.90)	0.94 (0.76-1.15)
Unknown	43 (2.6)	7061 (2.47)		1.03 (0.76-1.41)	0.78 (0.51-1.19)

*HS*, Hidradenitis suppurativa; *OR*, odds ratios.

∗ *P* values correspond to the χ^2^ analysis of the respective demographic factor for HS compared with non-HS cohorts.

† Multivariable regression controlled for all socioeconomic and lifestyle factors listed in Tables I and II.

‡ Defined as >100 cigarettes in their lifetime.

We observe a higher prevalence of HS in women and in SOC, although there was not a higher odds of HS in Hispanics. Our data support mixed findings regarding HS risk in SOC; some studies finding high rates in Blacks (1.3%) versus Hispanics (0.7%).^2^ Recent studies have suggested HS may be associated with lower socioeconomic status, and our analysis finds a higher risk of HS in lower income patients.^3^ However, we find a lower prevalence of HS in uninsured, unemployed, and less educated patients, which may reflect an underdiagnosis because of reduced health care access in these populations. A higher odds of disability in patients with HS may reflect a casual or associative relationship.

We find a higher OR of HS in nonbinary patients, a finding supported by recent literature, however our small sample size limits broad conclusion making.^4^ Smoking is an established risk factor for HS, and to our knowledge, we are among the first studies to observe a higher odds of HS in patients with e-cigarette (including vaping), and hookah users. The underlying pathophysiology is likely similar to smoking; nicotine/tobacco may promote proinflammatory cytokine release and keratinocyte, fibroblast, and immunocyte activation.^5^

Limitations to the study include the All of Us database uses a nonrepresentative sample of the US population and is limited to patients sharing electronic health record data. SOC, women, nonbinary, lower socioeconomic status, and smokers, all experience higher odds of HS. Timely identification of susceptible populations can decrease diagnostic delay, facilitate early treatment, and guide preventive and screening initiatives.

## Conflicts of interest

Dr Rao is a speaker for Incyte. The other authors have no conflicts of interest to declare.
